# Skin Barrier Dysfunction in Chronic Dermatoses: From Pathophysiology to Emerging Therapeutic Strategies

**DOI:** 10.7759/cureus.86937

**Published:** 2025-06-28

**Authors:** Irisdey Espinoza Urzua, María Isabel Vidal Vidal, Manrique Vega Solano, Julian Eduardo Bedoya Jaramillo, Gifneth Giselle de la Cruz Donis, Andres Romero Valverde

**Affiliations:** 1 Internal Medicine, Universidad Nacional Autónoma de México, Mexico City, MEX; 2 Health Education, Universidad Católica de El Salvador, Santa Ana, SLV; 3 Emergency Medicine, Vegas Medical Group, San José, CRI; 4 Internal Medicine, Clínica Andes Salud El Loa, Calama, CHL; 5 General Practice, Universidad San Carlos de Guatemala, Guatemala City, GTM; 6 Plastic and Reconstructive Surgery, Caja Costarricense de Seguro Social, San José, CRI

**Keywords:** advanced treatment, dermatoses, dysfunction, skin barrier, therapeutic strategies

## Abstract

Multidisciplinary investigations have confirmed the critical role of skin barrier dysfunction in the pathogenesis of various chronic dermatoses. However, the heterogeneity in disease presentation, inconsistent assessment criteria, and the lack of therapeutic standardization across clinical settings limit the universal applicability of current findings. The aim of this review is to evaluate the pathophysiological basis of skin barrier impairment in chronic skin conditions, assess the clinical efficacy of emerging therapeutic strategies, and address ongoing challenges and future directions. Skin barrier dysfunction underlies the initiation and perpetuation of inflammatory dermatoses such as atopic dermatitis, psoriasis, and ichthyoses, where disruptions in lipid composition, filaggrin deficiency, and impaired tight junction integrity contribute to disease chronicity. Innovative treatment approaches, including targeted biologics, barrier-repair emollients, and microbiome-modulating therapies, have demonstrated encouraging results in restoring barrier function and controlling inflammation. Emerging nanotechnological and gene-editing therapies offer promising frontiers for precision skin repair. Evidence supports that restoration of the barrier not only alleviates clinical symptoms but also reduces flare frequency and improves patient quality of life. Despite progress, therapeutic implementation faces obstacles such as patient adherence variability, lack of long-term outcome data, and inter-study inconsistencies in outcome measures. This review emphasizes the necessity of standardized protocols and unified barrier assessment tools to enhance clinical translation. The growing body of evidence advocates for integrating barrier-targeted strategies as a central element in the management of chronic dermatoses, thereby improving disease control and overall dermatological care.

## Introduction and background

This literature review is conducted to critically examine recent advances and limitations. This review aims to support the development of more precise, long-lasting, and patient-centered treatment strategies. Treating skin barrier problems may lead to improved care and better lives for patients and also suggests preventative and precise ways to help people with chronic skin diseases. Skin is the largest body part; it helps protect our internal organs from outside triggers [1]. There are three main layers: the epidermis, dermis, and hypodermis. Keratinocytes in the epidermis create a covering that holds in water, and the dermis, which is made of thick connective tissue, is home to blood vessels, nerves, sweat glands, and hair follicles. Adipose tissue in the hypodermis rebounds external pressure, conserves energy, and protects the body [2-4]. The outermost layer of the skin, mainly made up of the stratum corneum, lipids, and proteins, is called the skin barrier [5]. It supports hydrated skin by limiting moisture loss and functions as a primary barrier against germs, allergens, pollution, and irritants [6]. The skin’s health relies heavily on the strength of its barrier [7]. It balances the hydration of your skin, interacts with your immune system, and shields your body from foreign invaders. If this barrier is disrupted, it triggers many more linked responses in the skin that can cause skin diseases [8].

When the outer layer of the skin cannot defend properly, it is said to have a dysfunctional barrier [9]. This phenomenon may be caused by mutations, changes in the environment, invasion by microbes, or an inflammatory state [10]. When the skin is dysfunctional, it becomes permeable, often feels very dry, has weakened immunity, and allows more irritants and allergens in, which can influence the growth and worsening of chronic dermatological illnesses [11]. Various chronic diseases of the skin, some of which are tough to control, are referred to as chronic dermatoses [12]. Atopic dermatitis (AD) is known to result from reduced filaggrin and disrupted lipids, which compromise the skin barrier [13]. The cells in psoriasis increase and are not robust enough, which breaks the skin’s integrity [14]. Another chronic dermatosis is called ichthyosis vulgaris (IV), which leads to dry, flaky skin due to a dysfunctional shedding process of the skin [15]. Netherton syndrome (NS) is a rare disorder that brings together symptoms of ichthyosis, strange-looking hair, and a tendency to suffer from atopic problems [16]. Also, seborrheic dermatitis (SD) comes back and affects regions of the body with a lot of sebaceous glands, for example, the scalp and face [17].

In clinical care, problems with the skin barrier cause disruption to the skin as well as make people more likely to have repeated infections, experience increased pain, and struggle with mental and social issues [18,19]. Most patients develop itchiness, scales, redness, and discomfort, negatively impacting their daily lives [20]. When the skin barrier does not function well over a long period, patients may experience sleeping troubles, stress, sadness, and isolation from others. Because of this, treating barrier dysfunction requires management by various healthcare professionals [21]. Since skin barrier failure leads to chronic skin conditions, many therapies are now working on repairing and rebuilding the skin barrier [22]. Researchers are looking into using filaggrin inducers and protease inhibitors to treat the underlying causes of atopic dermatitis and Netherton syndrome [23]. Treatments that affect IL-4, IL-13, and IL-17 cytokines are proving useful at restoring the body’s conditions [24]. Besides, researchers are studying topical probiotics and microbiota transplants to help restore the skin’s usual protection [25]. Because of them, the method of treating inflammatory diseases has switched from conventional therapy to one that addresses the skin barrier and provides more lasting and disease-modifying results with fewer side effects [26].

This review is especially timely given the growing challenges faced in dermatological care. Skin disorders are being diagnosed at higher rates than ever before, affecting patients across all age groups and regions. This rise is not only creating a heavier demand on healthcare services but is also revealing shortcomings in how these conditions are managed [27]. Many of the current treatments provide only short-term relief and do not fully address the biological drivers of disease progression. As a result, patients often face recurring symptoms and fluctuating responses to therapy [28]. Moreover, the wide variability in how individuals respond to treatment highlights a major gap in personalized and effective management plans.

## Review

Methodology

This literature review explores the underlying mechanisms and therapeutic advances in skin barrier dysfunction associated with chronic dermatoses. A structured search of English-language, peer-reviewed articles was performed using databases including PubMed, ScienceDirect, and Google Scholar. Search terms included combinations of keywords such as "skin barrier dysfunction," "chronic dermatoses," "Atopic Dermatitis," OR "Psoriasis," OR "Ichthyosis Vulgaris," OR "Netherton syndrome" OR "Seborrheic Dermatitis," AND "pathophysiology" OR "EASI score" OR "patient-reported outcomes" OR "PASI response" OR "symptom reduction" OR "quality of life improvement" and "emerging therapies," using Boolean operators (AND, OR) to refine results. Articles were selected based on relevance and recent publication, preferably within the last five years, between January 2011 and 30 May 2025.

Results and discussion

Interlinked Pathophysiology

The skin barrier in the stratum corneum and tight junctions is crucial for keeping the skin hydrated and protecting it from allergens and microorganisms. According to Deng et al., chronic dermatoses can lead to skin barrier dysfunction due to genetic, immunological, and environmental factors. [29]. Konya et al. suggested that AD is caused by decreased function in the filaggrin gene, which affects keratin binding and natural moisturizing factor production, leading to increased transepidermal water loss (TEWL). Low levels of ceramides among lipids weaken the barrier function. Staphylococcus aureus colonization intensifies inflammation and further harms the protective barrier [30]. According to Liu et al., psoriasis occurs due to rapid multiplication of keratinocytes, damaging the skin barrier and causing immature corneocytes to form improperly. The immune system secretes chemicals like IL-17, IL-22, and TNF-α to disrupt the skin barrier. A decrease in lipid ceramides worsens the skin's ability to protect itself [31].

Sakai and Hatano suggested that ichthyosis vulgaris is caused by mutations in the FLG gene, leading to poor cornification and drier skin. Netherton syndrome, a rare disease, causes LEKTI, a serine protease inhibitor, to not function properly, leading to overwork and destruction of desmoglein-1, causing skin detachment too early, severe inflammation, intense redness, flaking, and susceptibility to allergies [32]. Santos indicated that seborrheic dermatitis is caused by an overgrowth of *Malassezia* yeast, which produces free fatty acids that trigger an inflammatory response. Overly sensitive sebaceous glands and an overactive immune system can disrupt skin renewal and weaken the skin barrier in areas with high oil content. These conditions often involve poor keratinocyte differentiation, unbalanced lipids and proteins, increased water loss, and hyperactive immune processes [33].

Sun et al. said that the epidermis contains the skin barrier, and its outermost area, the stratum corneum, is what primarily safeguards the skin. It contains mature cells, known as corneocytes, as well as a lipid matrix. Sometimes, people describe the skin barrier as a structure with corneocytes as bricks and lipids such as ceramides, cholesterol, and free fatty acids as mortar [34]. Abreu et al. concluded that due to these lipids, the layers separating corneocytes shield the body from water loss and prevent allergens, bacteria, and toxins from entering. Filaggrin, loricrin, and involucrin are needed to keep the upper layer of the skin strong and reliable [35].

Briganti et al. and Campbell et al. indicated that sometimes, problems with the skin barrier arise due to genetics and the environment. Filaggrin mutations have been extensively studied because they prevent keratinocytes from forming a suitable skin barrier. It is important for skin moisturization because it encourages the formation of strong keratin fibers and then converts itself into natural moisturizers. If the filaggrin protein is absent or altered, the skin loses moisture, making it easier for potential irritants to affect it [36,37]. Furthermore, Cho et al. and De Simoni et al. showed that high ceramide levels in the skin’s lipids make the barrier function weaker and allow more substances to go through, entering the body. The quality of the tight junctions between keratinocytes is necessary to oversee paracellular transport. When claudins and occludins are damaged, they cannot properly seal the skin and allow more allergens and bacteria to enter. Damage to the barrier worsens due to external factors like harsh soaps, low humidity, UV rays, and the development of bacterial colonies [38,39].

Gu et al. concluded that besides causing tissue damage, inflammation often contributes to chronic skin diseases by combining forces with problems in the skin’s barrier. When someone has atopic dermatitis, changes in filaggrin and skin lipids allow allergens to enter the skin and lead to a Th2-mediated reaction. Reduced filaggrin and lipids continue inflammation in the skin, repeating the damage done to the barrier [40]. Hoshino et al. found that even though psoriasis is mainly caused by rapid skin cell growth and disturbances in immune activity, problems with the skin barrier occur in most people struggling with the condition. IL-17 and TNF-alpha signal the skin’s building blocks (keratinocytes) to behave poorly, hurting the skin’s protective mechanism [41]. Sivori et al. and Türkoğlu et al. concluded that if a person has ichthyosis, mutations in keratinocytes and fats in the skin cause the skin to thicken and become scaly, resulting in a weak skin barrier. When the barrier is disrupted and microbes attack for a long period, it usually leads to secondary chronic inflammation. All in all, when a barrier becomes faulty, inflammation can worsen and vice versa, and this back and forth causes serious, ongoing dermatological conditions [42,43].

Emerging therapeutic strategies

Atopic Dermatitis

IL-13 inhibitors (biologics): Treating with IL-13 blockers is effective in managing atopic dermatitis that is moderate to severe. Guttman-Yassky et al. concluded that among all doses, lebrikizumab reduced Eczema Area and Severity Index (EASI) scores the most at week 16; at the highest dose (250 mg every two weeks), EASI scores dropped by 72.1%, while the same scores dropped only 41.1% with placebo [44]. The pruritus and sleep disturbance symptoms improved. Blauvelt et al. indicated that cendakimab reported efficacy at a 720 mg dose by showing an 84.4% reduction in EASI scores [45]. According to Magnolo et al., IL-13 remains a suitable target for biologic therapy in AD and encourages the creation of IL-13 inhibitors that are safe to use [46].

JAK inhibitors (oral and topical): Due to their power to block various cytokine messages related to AD symptoms, JAK inhibitors are an area of quickly emerging drugs for AD treatment. Magnolo et al. suggested that upadacitinib, an oral JAK1-selective inhibitor, combined with topical corticosteroids (TCS), reported efficacy within one to two weeks, sustained over a 52-week period by showing rapid improvements in itch, skin pain, and quality of life [46]. Wollenberg et al. indicated that over a 3.6-year period, baricitinib was found to keep clear or almost clear skin visible in almost 56% of children [47]. Lé and Torres concluded that among adolescents and adults, applying ruxolitinib to their skin showed good results and significant decreases in itch (half experienced a ≥4-point reduction in their Numeric Rating Scale (NRS) score) and skin lesions, with the improvement being maintained for up to an entire year [48]. Cai et al. suggest that JAK inhibitors offer quick results and address several issues in AD, such as inflammation, itch, and damage to the skin barrier [49].

Microbiome-targeted therapies: The skin microbiome supports both the skin barrier and the immune system in patients with atopic dermatitis. Some studies have found that people with AD frequently do not have enough of the strains *S. epidermidis* and *S. hominis *among coagulase-negative staphylococci (CoNS), which protect against *S. aureus.* According to Nakatsuji et al., when applied to the skin, antimicrobial strains helped restore the balance of skin microbes and reduce *S. aureus* colonization in both animals and humans [50]. Other research, carried out by Shimamori et al., showed that adding a *(SaGU1)* bacteriophage to *S. epidermidis* reduced both the number of *S. aureus* and inflammation in murine models of AD. These approaches for restoring the microbiome provide hope and a new option for treating AD in patients who have frequent infections or imbalances in their microbiomes [51].

OX40/OX40L pathway inhibitors:** **AD is linked to chronic inflammation since the OX40-OX40L signaling pathway plays a role in making T-cells active and forming memory, which is linked to this disease. Lé and Torres concluded that after a person stopped receiving the treatments, rocatinlimab and amlitelimab continued to help the patients for a longer period. After receiving the drug for 16 weeks, EASI scores went down by up to 61.1%, and this effect continued for another four weeks after the treatment finished [48]. According to Abdelhalim et al., patients treated with amlitelimab experienced a reduction of up to 80.1% and an extended response for as long as 68% at 24 weeks. They are effective because they extend the period of remission and reduce the usual requirement for constantly suppressing the immune system [52].

Allergen-specific immunotherapy: Sublingual immunotherapy (SLIT) is a method that targets those with AD and allergies to house dust mites. Liu et al. conducted a trial with *D. farinae* drops on patients with AD caused by mites. Both groups receiving the higher doses saw improved SCORing Atopic Dermatitis (SCORAD) scores and a decrease in their skin lesions and medicine consumption. SLIT did not cause any major problems for the patients. For patients with allergy sensitization, this type of immunotherapy gives a way to alter their immune system for a long time without using drugs that suppress the immune system [53].

Psoriasis

TYK2 inhibitors (oral small molecules): Oral TYK2 inhibitors are modern drugs that target the immune system, with fewer side effects compared to older and broader immunosuppressed drugs. Strober et al. concluded that deucravacitinib, a selective TYK2 inhibitor, reported efficacy within one to two weeks, sustained over a 52-week period, and showed good safety and tolerability [54]. Armstrong et al. highlighted that there were some increases in acne and folliculitis, but there were no serious issues or abnormalities found [55]. Jiang et al. concluded that, just like baricitinib, zasocitinib showed that the higher its dose, the more Psoriasis Area and Severity Index (PASI) 75/90/100 responses were reached in the first 12 weeks of treatment. PASI 100 at a dosage of 30 mg improved outcomes in 33% of patients [56]. They are a promising type of medicine that patients can take by mouth with reasonable safety considerations.

IL-17 inhibitors (biologics): Ansai et al. claimed that in Phase III, xeligekimab, an anti-IL-17A drug, was found to be very effective, and 90.7% of patients had achieved PASI 75 by week 12 and further improved during week 52. It appeared to be well tolerated, and nothing unexpected happened related to its safety [57]. In addition, Jiang et al. stated that IL-17 biologics remained effective even among patients who had inflammation related to metabolic syndrome. The study proves that IL-17 inhibition is an effective and proven way to treat psoriasis [56].

IL-23 inhibitors (biologics): Eyerich et al. investigated that inhibitors of IL-23 are another therapy used to control various diseases for the long term [58]. Peña-Corona et al. concluded that guselkumab, a type of IL-23 p19 subunit inhibitor, is the best dosage strategy. When treating super responders, week 16 was equally as effective as week eight in keeping the disease under control [59]. Furthermore, as reported by Jiang et al., IL-23 inhibitors can be beneficial for patients with any type of inflammatory syndrome. Because IL-23 inhibitors maintain their effectiveness for a long time, they are valuable for long-term care [56].

IL-12/23 inhibitors (biosimilars): With the advent of biosimilars, patients have access to a similar treatment at a lower cost. A 52-week study by Blauvelt et al., using ABP 654, showed it was as effective as the ustekinumab brand when targeting IL-12/23 [60]. Magnolo et al. indicated that ABP 654 and the reference product had an 81.9% improvement on the PASI measure in week 12. The safety and immunogenicity of CP-0554 were similar, and the use of ABP 654 in the clinical trial did not adversely change the results. Because of these findings, ABP 654 is expected to open up more opportunities for patients to receive the biologic therapy they need [46].

Ichthyosis Vulgaris

Symptomatic therapies (moisturizers, keratolytics, retinoids):** **Signs and symptoms are managed mainly by treatment with drugs and other methods. Among these, you can use emollients, urea, lactic acid, and acitretin to treat hyperkeratosis and make the skin softer. Chulpanova et al. and Peña-Corona et al. conveyed that, although topical agents are used first since they are easy to obtain and cost less, enteral retinoids produce stronger effects but are restricted by problems such as dryness in the mouth area and could cause birth defects. Even though these treatments help us maintain cleanliness, they do not cure the condition [59,61].

Biologic therapies (targeted immunomodulation): Though ichthyosis is typically caused by genes, abundant inflammation is seen in the severe forms of the condition. In their study, Yogarajah et al. reported that a patient with ABCA12-deficiency-related autosomal recessive congenital ichthyosis (ARCI) experienced a 48% decrease in Ichthyosis Area Severity Index (IASI) within six months after starting to use secukinumab, an IL-17A inhibitor [62].

Gene and cell therapies (curative prospects): Enhanced therapies are meant to address the main genetic factors behind ARCI and other kinds of ichthyoses. Chulpanova et al. and Joosten et al. state that using gene and protein therapies in the future may be beneficial. The purpose of these molecular therapies is to restore protein function, such as TGM-1 or ABCA-12, which could lead to normal barrier function being restored in the epidermis [61,63]. Cai et al. further added to the findings that even if they are still in early stages of testing, these approaches may see common use as their technology improves and they are found to be safe. The ability of these drugs to address the disease process instead of only its symptoms is a significant blessing [49].

Drug repositioning strategies: Peña-Corona et al. state that instead of developing new drugs, existing drugs and biologics can be used for ichthyosis [59]. Yogarajah et al. suggested that, among other things, you should also examine agents (for example, immunomodulators or metabolic modulators) that have already been approved to check if any of them can influence disease-related pathways. Though this approach is yet to be confirmed, it could eventually bring down the cost of treatments while using existing medicines. It is necessary to conduct detailed clinical exams to demonstrate that the medication is safe and effective for people with ichthyosis [62].

Novel immune targets (IL-18 blockade): Recently, scientists have been working on targeting certain cytokines that are linked to the worsening of the disease. Ansai et al. showed that IL-18 may play a role in epidermolytic ichthyosis (EI), since both the blood and skin samples of patients with EI had significantly higher levels of IL-18 and these levels depended on how severe the disease was [57]. Pontone et al.'s study was an analysis of biomarkers, but it implies that later research could focus on IL-18 inhibitors to help treat inflammation in EI [64]. Thus, doctors now recognize ichthyosis as a genetic condition that also has a lot to do with inflammation.

Netherton Syndrome

Biologic therapies (immune modulators):** **Biologic treatments are increasingly being explored in NS due to the immune dysregulation involved in its pathophysiology. IL-17A inhibitors, such as secukinumab, have shown the most consistent benefit. In a pediatric case series reviewed by Pontone et al. [64] and a separate case report by Mahajan et al. [65], IL-17A blockade led to reduced inflammation, pruritus, and skin scaling, along with downregulation of IL-17 expression and CD4+ Th17 cells. These outcomes support the hypothesis that NS shares inflammatory pathways with conditions like psoriasis and atopic dermatitis. Additionally, Truglio et al., targeting IL-12/23, IL-4R, IL-1β, and TNF-α, were reported to yield clinical improvement, though evidence is mostly anecdotal and lacks standardized protocols [66]. Despite the fact that they were tested using few volunteers, the safety of these two therapies has been good, suggesting they may be worthwhile after more testing in large groups.

Targeted protease inhibition (KLK5/KLK7 blockade): If someone has Netherton syndrome, it means they lack the LEKTI protein, which leads to KLK5 and KLK7 acting uncontrollably and causing the skin barrier to break and become inflamed. According to Chavarria-Smith et al., they invented a bispecific antibody meant to block both KLK5 and KLK7. Applying the antibody on mice with NS and AD increased the skin’s strength, reduced inflammation, and hindered skin flaking [67]. Based on the Tao et al. analysis, KLK5 activity was found to be inhibited allosterically, which suggests the strategy could lead to a mechanism-based therapy for this disease. Although at an early scientific stage, the results so far encourage moving closer to human trials [68].

Gene therapy (SPINK5 restoration): Gene therapy can be used to cure NS as it targets the gene responsible for PS1. Di et al. showed that restoring LEKTI with SPINK5, delivered by a lentiviral vector to patient cells grown in the lab, was successful [69]. According to Joosten et al., keratinocytes restored the structure of the skin in places adjacent to the cells that were only partially corrected. Still, this treatment method looks very promising for long-term skin repair and plays an important role in rebuilding the barrier, yet it must first be shown to be safe and effective for wider use [63].

Vaccine responsiveness and immune function: Given the risk of infection and concerns about immune competence in NS, Nouwen et al. assessed responses to conjugate (ActHiB), polysaccharide (PPV23), and mRNA COVID-19 vaccines. Results showed that patients generally mounted normal or near-normal humoral and T-cell responses, with no adverse events reported. While one patient showed no response to PPV23 and two had diminished responses, overall vaccine efficacy and immune responsiveness were within the healthy range. This reassures clinicians that vaccination remains safe and effective in NS and does not indicate a broad immunodeficiency [70].

Seborrheic Dermatitis

Topical non-steroidal anti-inflammatory agents (PDE-4 inhibitors): A promising recent addition to the topical therapy landscape in SD is roflumilast foam 0.3%, a phosphodiesterase-4 (PDE-4) inhibitor. In a Phase 3 clinical trial by Blauvelt et al., once-daily application over eight weeks resulted in significantly higher Investigator's Global Assessment (IGA) success rates (79.5%) compared to the vehicle group (58.0%), with benefits observed as early as week two. This therapy not only reduced inflammation effectively but also offered a favorable safety profile, with minimal local irritation [45]. Ansai et al. concluded that roflumilast represents a non-steroidal alternative suitable for long-term management of SD, although longer trials are needed to confirm sustained efficacy and safety in chronic use [57].

Microbiome-modulating treatments: Seborrheic dermatitis is well-known to be associated with microbial imbalance, particularly involving *Malassezia *species and *Staphylococcus*. Several studies explored microbiome-targeted treatments as emerging options. Truglio et al. investigated EUTOPLAC, an oily suspension containing probiotic strains (*Lactobacillus crispatus *and *Lacticaseibacillus paracasei*), applied for one week. Patients experienced symptom improvement and microbial rebalancing. *Malassezia *and *Staphylococcus *decreased, while beneficial bacteria increased [66]. Similarly, Tao et al. showed that ketoconazole 2% cream, a standard antifungal, not only reduced *Malassezia *but also enhanced fungal diversity, shifting the skin microbiome toward a healthier state [68]. These studies support the therapeutic potential of microbiome modulation, although limitations include short follow-up durations and small cohorts.

Immune trait-targeted research (genetic and diagnostic insight): A novel perspective on SD pathogenesis was provided by Xian et al. through a Mendelian randomization (MR) study using genome-wide association studies (GWAS) data. This genetic analysis identified 11 immune cell traits causally associated with SD, including Tregs, natural killer T (NKT) cells, and various B-cell subtypes [71]. Armstrong et al. concluded that some traits were linked to increased risk, while others were protective. The absence of heterogeneity or pleiotropy strengthens the credibility of these associations. While the present study is not a direct therapeutic strategy, it highlights immune traits as both diagnostic markers and future therapeutic targets, paving the way for more personalized, immune-modulating treatments in SD [55].

Clinical efficacy

Guttman-Yassky et al. and Simpson et al. suggested that the management of AD has seen significant advances with IL-13 inhibitors such as lebrikizumab and cendakimab showing strong, dose-dependent improvements in disease severity, itch, and quality of life [44,72]. Magnolo et al. and Wollenberg et al. found that JAK inhibitors like upadacitinib and baricitinib offer rapid and durable symptom relief, particularly for itch and inflammation, with favorable safety profiles [46,47]. Eichenfield et al. found that topical JAK inhibitors such as ruxolitinib cream provide effective localized treatment with minimal systemic exposure [73]. Lé and Torres and Nakatsuji et al. concluded that medicines aimed at the OX40/OX40L pathway appear to be useful for the long-term improvement of psoriatic disease by affecting T-cell inflammation, while new approaches to treating the microbiome are underway to enhance the skin barrier and function of immune cells [48,50].

Cai et al. [49] and Simpson et al. [72] concluded that skin clearance and durable remission rates are high for xeligekimab, guselkumab, and other IL-17 and IL-23 inhibitors used in plaque psoriasis. Strober et al. concluded that deucravacitinib is available as a safe and successful oral option [54]. Mahajan et al. concluded that IL-23 biologics could help treat metabolic co-diseases, and choosing biosimilars is a beneficial way to save money without increasing the risk of losing efficacy [65]. Xian et al. concluded that ichthyosis vulgaris is treated mostly with emollients and keratolytics that soften and moisturize the skin, and retinoid creams are only moderately beneficial, although gene therapies are currently still in the experimental stage [71]. Cai et al. suggested that even though retinoids can make a difference in Netherton syndrome by improving scaling and inflammation, they may have many side effects [49]. In contrast, Eichenfield et al. concluded that biologic treatments tackling Th2 and IL-17 pathways seem promising, and carefully managing the skin with regular treatments and infections is necessary because Netherton syndrome impairs the skin’s defenses [73].

Peña-Corona et al. and Truglio et al. indicated that for seborrheic dermatitis, antifungal agents like topical ketoconazole and ciclopirox remain first-line treatments targeting Malassezia species, supplemented by anti-inflammatory topical corticosteroids and calcineurin inhibitors to control inflammation and erythema, alongside medicated shampoos and emollients to maintain remission and scalp hygiene [59,66]. Shimamori et al. concluded that biologic medicines have made it simpler for experts to manage chronic skin problems, mostly atopic dermatitis and other inflammatory conditions [51]. Joosten et al. suggested that the monoclonal antibody lebrikizumab, which targets interleukin-13 (IL-13), has proven to be effective in reducing skin problems and symptoms in people suffering from moderate-to-severe atopic dermatitis during clinical trials [63].

Chavarria-Smith et al. suggested that for atopic dermatitis and eosinophilic esophagitis, cendakimab has proven to reduce skin inflammation and the number of skin lesions. They attack significant cytokines in the Th2 inflammatory pathway, so they work well for patients with asthma who do not benefit from traditional allergy drugs given by mouth or by inhaler [67]. Natarelli et al. suggested that biologics are more effective when patients stick with their treatment and respond differently to it. Adherence to injections may be affected by anxiety about getting them, the expense, people’s opinions about their effectiveness, and the side effects they may cause. Not every patient benefits from the same biologic treatment because their bodies react differently [74].

Characteristics of studies included in the review

Table 1 summarizes the characteristics and findings of the studies included in the review. It encompasses author/year, disease, treatment method, treatment dosage and duration, measured outcome, study findings, conclusion, and complication.

**Table 1:** Characteristics of studies included in the review AD: atopic dermatitis, ARCI: autosomal recessive congenital ichthyosis, EI: epidermolytic ichthyosis, NS: Netherton syndrome, SD: seborrheic dermatitis, EASI: Eczema Area and Severity Index, IGA: Investigator Global Assessment, NRS: Numeric Rating Scale, vIGA-AD: Validated Investigator Global Assessment Scale for Atopic Dermatitis, PASI: Psoriasis Area and Severity Index, PGA: Physician’s Global Assessment, SCORAD: SCORing Atopic Dermatitis, BSA: Body Surface Area, IASI: Ichthyosis Area Severity Index, CDLQI: Children’s Dermatology Life Quality Index, WI-NRS: Worst Itch-Numeric Rating Scale, POEM: Patient Oriented Eczema Measure, DLQI: Dermatology Life Quality Index, LD: loading dose, TCS: topical corticosteroids, QoL: quality of life, AE: adverse events, TEAE: treatment emergent adverse events, CoNS: coagulase-negative staphylococci, SLIT: sublingual immunotherapy, HDM: house dust mites, HDL-C: High-density lipoprotein cholesterol, CRP: C-reactive protein, ESR: erythrocyte sedimentation rate, MetS: metabolic syndrome, RP: reference product, IVIG: intravenous immunoglobulin, GWAS: genome-wide association studies, Q2W: once every two weeks, QD: every day, QOD: every other day

Author/Year	Disease	Treatment Method	Treatment dosage and duration	Measured outcome	Study findings	Conclusion	Complication
Guttman-Yassky et al. [44]	Atopic dermatitis	Lebrikizumab (IL-13 inhibitor)	125 mg every 4 weeks (250 mg LD), 250 mg every 4 weeks (500 mg LD), 250 mg every 2 weeks (500 mg LD at week 0 and 2), duration: 16 weeks	Primary: % change in the EASI score from baseline to week 16. Secondary: IGA 0/1; EASI-50/75/90; % change in pruritus NRS; ≥4-point NRS improvement	The study showed a significant reduction in EASI scores at 16 weeks, with a reduction of 41.1% in placebo, 62.3% in 125 mg, 69.2% in 250 mg, and 72.1% in 250 mg q2wk.	Lebrikizumab demonstrated rapid, dose-dependent efficacy and favorable safety in moderate to severe AD. The study supports IL-13 as a key target in the treatment of AD.	Mild-to-moderate adverse events. Injection site reactions: Herpes. Conjunctivitis.
Blauvelt et al. [45]	Seborrheic dermatitis	Phase 3 randomized, double-blinded, vehicle-controlled clinical trial	Adolescents and adults with SD (8-week trial)	IGA Success at week 8 (IGA score 0 or 1 + ≥2-point improvement from baseline); safety	79.5% of roflumilast group vs. 58.0% of vehicle group achieved primary outcome (P < .001); significant benefit also seen at weeks 2 and 4; well-tolerated	Roflumilast foam 0.3% was significantly more effective than vehicle for improving SD; favorable safety profile supports its use in clinical practice	Short trial duration (8 weeks) for a chronic condition; longer-term efficacy and safety need further study
Magnolo et al. [46]	Atopic dermatitis	Upadacitinib (oral JAK inhibitor) + TCS	Upadacitinib 15 mg or 30 mg once daily + TCS for 52 weeks	Patient-reported outcomes (itch, skin pain, sleep, QoL, daily activities, emotional state) at weeks 1-52	Significant improvements vs. placebo in PROs as early as 1-2 weeks (p < .05). At week 52, clinically meaningful improvements in itch (62.1%-77.7% at 15 mg; 71.3%-83.6% at 30 mg) and other symptoms were sustained	Upadacitinib + TCS leads to rapid and sustained improvements in itch, symptoms, and quality of life over 52 weeks	No specific complications reported in abstract; generally well tolerated
Wollenberg et al. [47]	Atopic dermatitis (Pediatric, age 2 to <18)	Baricitinib (oral selective JAK inhibitor)	Baricitinib 1 mg, 2 mg, or 4 mg equivalent; extended treatment up to 3.6 years	vIGA-AD scores at weeks 16 and 52	At week 52, 56.8% of baricitinib 4 mg responders maintained vIGA-AD 0/1 vs. 39.7% placebo. Safety: mostly mild/moderate AEs; no deaths or serious thrombotic or cardiovascular events reported	Baricitinib shows sustained long-term efficacy and favorable safety profile	No new safety signals; no serious adverse cardiovascular or thrombotic events
Lé & Torres [48]	Atopic dermatitis	Biologics targeting OX40/OX40L pathway: rocatinlimab (anti-OX40) and amlitelimab (anti-OX40L)	Rocatinlimab: Phase 2b, placebo-controlled trial (16 weeks + 20 weeks follow-up); Amlitelimab: Phase 2a, 12-week trial with extended follow-up	EASI percentage change from baseline; sustained response post-treatment	Rocatinlimab: -48.3% to -61.1% EASI reduction vs. -15.0% for placebo (p < 0.001); effects sustained 20 weeks post-treatment. Amlitelimab: -69.9% and -80.1% vs. -49.4% (p = 0.072 and p = 0.009); 68% of responders maintained benefit at 24 weeks post-treatment.	OX40-OX40L inhibitors show promise as disease-modifying therapies for moderate-to-severe AD, with prolonged effects and early response.	Both agents were well tolerated, with no major safety concerns reported.
Cai et al. [49]	Moderate-to-severe plaque psoriasis	Xeligekimab (GR1501), anti-IL-17A monoclonal antibody	Multicenter, randomized, double-blind, placebo-controlled Phase III: 12 weeks placebo-controlled + 40 weeks extension	PASI 75, 90, 100; PGA 0/1 at weeks 12 and 52	At week 12: PASI 75: 90.7% vs 8.6%; PASI 90: 74.4% vs 1.4%; PASI 100: 30.2% vs 0%; PGA 0/1: 74.4% vs 3.6%. Responses maintained to week 52	Highly effective and well tolerated in Chinese patients	No unexpected adverse events reported
Nakatsuji et al. [50]	Atopic dermatitis	Topical application of antimicrobial-producing commensal CoNS strains (e.g., S. epidermidis, S. hominis)	Laboratory screening, animal studies, and human pilot trials	S. aureus colonization levels, antimicrobial activity of CoNS strains, synergy with human AMPs (LL-37)	Antimicrobial CoNS strains were abundant in healthy individuals but rare in AD. These strains produced novel AMPs that selectively killed S. aureus. Application to mice and AD patients reduced S. aureus colonization.	Commensal CoNS strains with antimicrobial activity protect against S. aureus and are deficient in AD. Restoring these strains may be a therapeutic strategy for microbiome-targeted AD treatment.	No significant safety concerns reported; topical application well tolerated
Shimamori et al. [51]	Atopic dermatitis associated with Staphylococcus aureus	Microbiome-targeted therapies (Phage therapy using S. aureus phage SaGU1, combined with Staphylococcus epidermidis)	Topical administration on mouse back skin; specific dosage not stated; duration not detailed in abstract	S. aureus counts, IgE plasma concentration, histopathology, emergence of phage resistance	Significant reduction in S. aureus, IgE levels, and histological inflammation in treated groups	SaGU1 phage therapy can reduce S. aureus burden and improve AD; S. epidermidis prevents phage resistance	Not specifically reported
Abdelhalim et al. [52]	Atopic dermatitis	Biologics targeting OX40/OX40L pathway: rocatinlimab (anti-OX40) and amlitelimab (anti-OX40L)	Narrative review synthesizing trial data on both biologics	Skin clearance, symptom reduction, safety, disease severity	Both agents significantly outperformed placebo in reducing AD severity; favorable safety profiles; no serious treatment-related adverse events reported	OX40-OX40L inhibition is a promising strategy for moderate-to-severe AD, warranting further trials to assess long-term outcomes and safety	No serious treatment-related AEs reported
Liu et al. [53]	Atopic dermatitis	SLIT with D. farinae drops (HDM extract)	Multicenter, randomized, double-blind, placebo-controlled trial (36 weeks)	SCORAD index, total medication score, skin lesion area	Significant improvements in SCORAD and medication scores in medium/high-dose groups; skin lesion area reduced compared to placebo (p < .05)	SLIT with medium/high-dose D. farinae drops is effective and well-tolerated for HDM-induced AD	Mostly mild AEs; no life-threatening adverse reactions
Strober et al. [54]	Moderate-to-severe plaque psoriasis	Oral deucravacitinib (selective TYK2 inhibitor)	Pooled analysis of two phase 3 randomized trials (POETYK PSO-1 and PSO-2), 52 weeks	Safety and tolerability over 52 weeks	AE incidence similar across groups; serious AEs low and balanced; lower discontinuation rates with deucravacitinib; no new safety signals	Well-tolerated with acceptable safety	Some increased rates of acne and folliculitis with deucravacitinib; no major lab abnormalities
Armstrong et al. [55]	Moderate-to-severe plaque psoriasis	Oral zasocitinib (selective allosteric TYK2 inhibitor)	Phase 2b randomized, double-blind, placebo-controlled trial; 12 weeks treatment + 4 weeks follow-up	PASI 75, 90, 100 responses at week 12	Dose-dependent efficacy: PASI 75 achieved by 18%-68% (dose-dependent), placebo 6%; PASI 100 up to 33% at 30 mg	Potent, selective TYK2 inhibition with zasocitinib ≥5 mg improved skin clearance	TEAEs in 44%-62% (placebo 44%); no dose-dependency or lab parameter concerns
Jiang et al. [56]	Plaque psoriasis (psoriasis vulgaris) with/without metabolic syndrome	IL-17 biologics, IL-23 biologics, Cyclosporine (control)	Treatment lasted 3 months; assessments at baseline, 1 month, 3 months; dosing not specified	PASI score, blood glucose, triglycerides, HDL-C, CRP, ESR, IL-6, insulin	IL-17 & IL-23 groups showed significantly better PASI score and metabolic improvements vs control (P < 0.05); IL-23 superior at 3 months	IL-23 biologics recommended for long-term treatment in psoriasis patients with MetS due to better efficacy and metabolic improvements	Not reported
Ansai et al. [57]	Epidermolytic ichthyosis	Proposed therapeutic target: IL-18 blockade (novel suggestion based on biomarker study)	Not applicable (biomarker study)	Serum and skin IL-18 levels; Severity correlation (Ichthyosis Scoring System)	Serum IL-18 was significantly elevated in EI patients (2714.1 (1438.0) pg/mL vs. 218.4 (28.4) pg/mL; P < 0.01). IL-18 correlates with disease severity. IL-18 blockade suggested as novel therapy.	IL-18 blockade as a promising novel therapeutic approach for EI.	Not specified
Eyerich et al. [58]	Moderate-to-severe plaque psoriasis	Guselkumab (IL-23 p19 inhibitor) 100 mg	Phase 3b, randomized, double-blind, 68 weeks total (Part 1: 28 weeks induction, Part 2: 40 weeks maintenance)	Noninferiority of 16-week vs 8-week dosing for maintaining PASI <3 at week 68	PASI <3 maintained in 91.9% (16-week) vs 92.6% (8-week), meeting noninferiority margin (P=0.001)	Extending dosing interval to 16 weeks in super responders effectively maintains disease control	Well tolerated, no new safety signals
Peña-Corona et al. [59]	Autosomal recessive congenital ichthyosis	Topical treatments (moisturizers, keratolytics), enteral retinoids, drug repositioning strategies (potential biologics and drugs)	Not specified; topical is first choice; enteral retinoids offer greater efficacy but with limitations	Symptom relief, quality of life improvement	Symptom relief, quality of life improvement	Topical treatments are first-line due to ease and cost; enteral retinoids are more effective but limited by side effects. Drug repositioning offers potential affordable new therapies. ARCI remains lifelong and disabling despite current options.	Side effects from retinoids noted:
Blauvelt et al. [60]	Moderate-to-severe plaque psoriasis	ABP 654 (ustekinumab biosimilar) vs. ustekinumab RP	Weight-based dosing: 45 mg or 90 mg subcutaneous injection at day 1 (week 0), week 4, week 16; 52-week follow-up	Percentage improvement in PASI at week 12 (primary); safety and immunogenicity throughout 52 weeks	Mean PASI improvement at week 12 was 81.9% for both groups; differences were within pre-specified similarity margins. Safety and immunogenicity profiles were comparable. Transition from RP to ABP 654 at week 28 had no negative impact.	ABP 654 is clinically similar to ustekinumab RP in efficacy, safety, and immunogenicity for moderate-to-severe plaque psoriasis, supporting biosimilarity	No clinically meaningful safety or immunogenicity issues reported
Chulpanova et al. [61]	Autosomal recessive congenital ichthyosis and other inherited ichthyosis types	Symptomatic therapy: moisturizers, keratolytics, retinoids, cosmetic substances; graft transplantation for eye defects; Emerging gene and cell therapies	Not specified	Improvement in skin condition; correction of protein function (prospective)	Improvement in skin condition; correction of protein function (prospective)	Symptomatic therapies improve skin condition but do not restore protein function or provide long-term skin barrier. Gene and cell therapies are promising to correct protein function but remain at an early research stage.	Not specified
Yogarajah et al. [62]	Severe autosomal recessive congenital ichthyosis (ABCA12 deficiency-related)	Secukinumab (IL-17A inhibitor)	6-month trial: 150 mg subcutaneous weekly injections for 4 weeks (induction), then 150 mg every 4 weeks for 20 weeks (maintenance)	IASI reduction; cytokine profile changes	48% reduction in IASI score after 6 months; reduction in proinflammatory cytokines and Th17 skewing; treatment well tolerated; suggests IL-17A inhibition is promising	A 48% IASI reduction and decreased proinflammatory cytokines suggest IL-17A inhibition as a promising and well-tolerated treatment for ichthyosis.	No adverse effects reported; treatment well tolerated
Joosten et al. [63]	Ichthyosis spectrum, including autosomal recessive congenital ichthyosis, Netherton syndrome	Pathogenesis-based therapies: protein replacement therapy, gene therapy, biological therapeutics	Various ongoing trials, dosage/duration not specified	Efficacy of molecular and biological therapies in improving skin symptoms	Efficacy of molecular and biological therapies in improving skin symptoms	Molecular therapies and biologicals show promise; several ongoing trials; biological therapeutics effective, especially for Netherton syndrome and ARCI; expected to become part of standard treatment	Not specified
Pontone et al. [64]	Netherton syndrome (Pediatric)	Biologics: anti-IL-17A, anti-IL-12/23, anti-IL-4R/IL-13R, anti-TNF-α, anti-IL-1β; also IVIG	Case reports, no standardized regimen or dosage	Clinical response: reduction in skin inflammation, IgE levels, allergic manifestations	Biologic therapies, especially IL-17 blockade, showed clinical improvement and were generally well tolerated; IVIG also showed benefit, limited by small sample sizes and absence of placebo controls. IL-17/IL-36 pathways are key targets; future treatments may include barrier repair plus biologics; further controlled trials needed	IL-17 blockade and IVIG demonstrated clinical improvement and tolerability in ichthyosis, highlighting IL-17/IL-36 as key targets, though further controlled trials are needed.	No severe side effects reported
Mahajan et al. [65]	Netherton syndrome	Anti-IL-17A therapy (subcutaneous secukinumab)	Not fully detailed; 6-month treatment duration	Clinical response, IL17A gene expression, CD4+ Th17 cell population	Favorable clinical response with reduced pruritus and scaling; downregulation of IL17A expression and CD4+ Th17 cells after 6 months, showing abrogation of Th17-skewing. Well tolerated.	Treatment led to reduced pruritus and scaling with downregulation of IL17A and Th17 cells, supporting effective and well-tolerated Th17 pathway modulation.	None reported
Truglio et al. [66]	Seborrheic dermatitis	Interventional cohort study with microbial and clinical assessments	25 SD patients	Symptom severity, skin mycobiome and bacteriome changes at baseline (T0), 7 days (T8), and 21 days (T28) post-treatment	Improved symptoms; ↓ Malassezia and Staphylococcus; ↑ Lactobacillus and Lacticaseibacillus; altered microbial network with more negative correlations post-treatment	EUTOPLAC may serve as a microbiome-modulating treatment for SD, showing clinical improvement and microbial shifts consistent with reduced inflammation and dysbiosis	Small sample size; short treatment and follow-up duration; no control group or blinding
Chavarria-Smith et al. [67]	Netherton syndrome and atopic dermatitis	Bispecific antibody targeting KLK5 and KLK7 (murine and humanized forms)	Not specified (preclinical model)	Skin barrier function, inflammation, desquamation control	Bispecific anti-KLK5/7 antibody improved skin barrier integrity and reduced inflammation in NS and AD mouse models. Structural studies show allosteric inhibition of KLK5. Promising for clinical development.	Bispecific anti-KLK5/7 antibody enhanced skin barrier function and reduced inflammation, showing promise for clinical development in NS and AD.	Not reported (preclinical)
Tao et al. [68]	Seborrheic dermatitis	Prospective cohort with microbial community sequencing	30 facial SD patients + 15 healthy controls	Skin bacterial and fungal diversity and community composition before, immediately after, and 2 weeks post-treatment	Facial SD had lower bacterial/fungal diversity, ↑ Malassezia and Staphylococcus, ↓ Cutibacterium; Ketoconazole ↓ Malassezia, ↑ fungal diversity (Candida, Aspergillus), lasting 2 weeks post-treatment	Ketoconazole treatment modulates fungal microbiome, reducing Malassezia and restoring microbial diversity in SD lesions	Limited follow-up duration; no placebo control; only facial SD evaluated
Di et al. [69]	Netherton syndrome	Ex vivo gene therapy using lentiviral vector delivering SPINK5 to keratinocytes	Single application; duration not specified; evaluated in vitro and in vivo (skin graft model)	LEKTI expression, epidermal architecture, bystander effect	High transduction efficiency and restoration of LEKTI in NS keratinocytes; corrected architecture in both organotypic cultures and humanized mouse grafts. Notably, even partial LEKTI restoration had bystander benefits. Potential for therapeutic benefit in patients.	Restoration of LEKTI improved epidermal architecture with bystander effects, supporting its therapeutic potential for Netherton syndrome.	None reported
Nouwen et al. [70]	Netherton syndrome	Vaccines (Polysaccharide PPV23, Conjugate ActHiB, and mRNA COVID-19 booster)	Cross-sectional study (Jan–Aug 2022); single-dose vaccine responses analyzed; booster mRNA COVID-19 vaccine given	Antibody levels and T-cell responses to vaccines	ActHiB induced normal responses in 7/7; PPV23 responses: 1/7 absent, 2/7 diminished, 4/7 normal; SARS-CoV-2 antibody and CD4+ T-cell responses comparable to controls; CD8+ response detectable in 2/6; overall, vaccine responses within the spectrum of healthy population and no signs of clinically significant B/T-cell immunodeficiency	Vaccine responses in NS patients were largely comparable to healthy controls, indicating no clinically significant B- or T-cell immunodeficiency.	No adverse events reported
Xian et al. [71]	Seborrheic dermatitis	Two-sample bidirectional Mendelian Randomization (MR) study using GWAS data	GWAS Catalog: 731 immune cell traits as exposures; SD as outcome	Causal association with risk of SD	Identified 11 immune cell traits causally linked to SD: 5 associated with lower risk, 6 with higher risk. No evidence of heterogeneity or pleiotropy.	Immune traits have causal roles in SD pathogenesis and may serve as diagnostic biomarkers or therapeutic targets.	None reported
Simpson et al. [72]	Mild-to-moderate AD (≥12 yrs, adults & adolescents)	Ruxolitinib cream (JAK1/JAK2 inhibitor), topical	0.75% or 1.5% cream, twice daily for 8 weeks (VC), then as-needed up to 52 weeks (LTS)	PROs: Itch (POEM), skin pain (NRS), sleep (POEM, PROMIS), QoL (DLQI/CDLQI)	Significant improvements in skin pain within 12 hours were observed, with POEM, DLQI, and CDLQI all showing a p-value of less than 0.0001 compared to vehicle.	Ruxolitinib cream rapidly improved itch, skin pain, sleep, and QoL, with benefits sustained through 52 weeks	Well tolerated; no new safety signals reported
Eichenfield et al. [73]	Atopic dermatitis (Adolescents 12-17)	Ruxolitinib cream (JAK1/JAK2 inhibitor) topical	1.5% cream, 8 weeks continuous, then up to 52 weeks as-needed	IGA Treatment Success (IGA 0/1 + ≥2-grade improvement), EASI-75, Itch NRS reduction	At week 8, the patient experienced significant improvement in IGA-TS, EASI-75, and NRS4 itch, demonstrating sustained disease control over 52 weeks.	Significant anti-inflammatory and antipruritic effects with sustained disease control during long-term as-needed use	Low rate (1.8%) of application site reactions; no serious AEs, infections, malignancies, cardiovascular or thromboembolic events
Natarelli et al. [74]	Acne, psoriasis, AD	Bacteriophage (phage) therapy / Phage-replacement therapy	Not specified (review article)	Control of pathogenic bacterial overgrowth; reduction in dermatologic disease symptoms	Phage therapy shows promise in reducing bacterial overgrowth and improving symptoms in dermatologic diseases, but current studies are limited	Phage therapy is a promising approach in dermatology, but larger, controlled trials are needed to determine optimal delivery, dosage, and efficacy	Not reported
Blauvelt et al. [75]	Atopic dermatitis	Cendakimab (IL-13 inhibitor)	360 mg every 2 weeks; 720 mg every 2 weeks; 720 mg once weekly; all subcutaneously for 16 weeks	Mean % change in EASI score from baseline to week 16	720 mg once weekly: −84.4% vs −62.7% (P = .003); 720 mg q2wk: −76.0% (P = .06); 360 mg q2wk: −16.3% (nominal P = .03); however, significance for 360 mg not claimed due to hierarchy testing protocol being interrupted.	Cendakimab (particularly 720 mg weekly) showed significant efficacy and was generally safe and well tolerated over 16 weeks.	TEAEs leading to discontinuation: 7.4% (720 mg weekly), 3.6% (720 mg q2wk), 1.8% (360 mg q2wk), 3.6% (placebo); overall tolerable safety profile.
Simpson et al. [76]	Atopic dermatitis	Lebrikizumab (IL-13 inhibitor)	250 mg subcutaneously every 2 weeks for 16 weeks (monotherapy)	% change in EASI, Pruritus NRS, Sleep-Loss Scale, POEM, DLQI at 16 weeks	Significant improvements in ADvocate1/ADvocate2 were observed in EASI, Pruritus NRS, Sleep-Loss Scale, POEM, and DLQI, with all improvements being significant at each measured time point.	Lebrikizumab monotherapy produced rapid, consistent, and significant improvements in clinical signs, symptoms, and QoL over 16 weeks.	no major safety concerns were highlighted.
Blauvelt et al. [24]	Moderate-to-severe AD in adolescents (12–17 years)	Dupilumab (subcutaneous monoclonal antibody)	Initially: 2 or 4 mg/kg weekly; then 300 mg every 4 weeks; uptitrated to 200 mg (<60 kg) or 300 mg (≥60 kg) every 2 weeks if inadequate response; treatment duration: up to 52 weeks	- IGA 0/1 (clear/almost clear skin) - EASI-50/75/90 - SCORAD - BSA - CDLQI - Safety via TEAEs, SAEs, lab values, re-initiation data	- 42.7% achieved IGA 0/1 by week 52- 93.1% achieved EASI-50, 81.2% EASI-75, and 56.4% EASI-90- 70.9% required uptitration to q2w regimen- 29.4% sustained IGA 0/1 over 12 weeks and stopped treatment; 56.7% of them relapsed- Uptitration improved response rates and EASI scores	Dupilumab showed sustained efficacy and acceptable safety over 52 weeks in adolescents. The q2w regimen is optimal, as most patients needed uptitration. Continuous treatment is generally required to maintain skin clearance.	- 73.8% experienced ≥1 TEAE (mostly mild/moderate)- Serious TEAEs in 1.7%- Conjunctivitis in 8.8%, injection-site reactions in 6.5%, herpes virus infections in 6.1%- 2 patients discontinued due to adverse events (conjunctivitis, worsening AD)
Simpson et al. [77]	Atopic dermatitis	Topical application of roflumilast cream (0.15%)	0.15% cream once daily for 4 weeks	- vIGA-AD Success (primary) - EASI score - WI-NRS - Local tolerability and safety	- At week 4, 31.3% roflumilast vs. 14.1% vehicle achieved vIGA-AD Success (P<0.0001) - 92% showed any improvement in EASI by Week 4 - EASI-50: 69.2% vs. 44.4% - EASI-75: 44.5% vs. 21.2% - EASI-90: 22.4% vs. 8.6% - EASI-100: 9.8% vs. 4.8% (all P<0.0001)	Roflumilast cream 0.15% significantly improved EASI, vIGA-AD, and WI-NRS outcomes with rapid onset and good tolerability in AD patients over 4 weeks.	Very low rate of adverse events; >90% patients had no or mild sensation at application site. No significant complications reported.
Miguel Nogueira et al. [78]	Psoriasis & psoriatic arthritis	Oral JAK inhibitors (focus on selective TYK2 inhibitors)	Varies by drug: Tofacitinib: 5–10 mg twice a day BMS-986165: 3 mg QOD to 12 mg QD (12-week duration) Brepocitinib: 30–100 mg QD (4–12 weeks)	PASI75, PGA, ACR20, safety profiles, AE), laboratory parameters, and durability of response	- Tofacitinib: PASI75 in 40–64% of patients; non-inferior to etanercept; maintained efficacy for 2 years; higher dose associated with more risk. - BMS-986165: PASI75 in up to 75% of patients; safe and well tolerated; common AEs were nasopharyngitis, headache, nausea; no herpes zoster or cardiovascular events. - Brepocitinib: PASI75 up to 86.2%; mild-to-moderate AEs; one death (unrelated); dose-related platelet and reticulocyte count reduction observed.	Selective TYK2 inhibitors (esp. BMS-986165) show a promising balance between efficacy and safety for oral psoriasis treatment. Tofacitinib effective but with safety concerns.	- Tofacitinib: Risk of herpes zoster, gastric perforation, and thromboembolic events (especially at higher doses) - Brepocitinib: Platelet/reticulocyte suppression - BMS-986165: No major complications reported in trials

Challenges

Although new targeted therapies have been developed for chronic dermatoses, some problems still exist. The fact that AD can be different for each patient and that response to treatment is not always the same means doctors have trouble finding the most suitable therapies. Simpson et al. stated that long-term safety data for biologics and JAK inhibitors are also not yet available [72]. Jiang et al. suggested that biologic medicines for psoriasis are very expensive and finding individual treatments for comorbidities like metabolic syndrome is a challenge [56]. Chulpanova et al. indicated that in cases of rare genodermatoses, specialized forms of ichthyosis vulgaris and Netherton syndrome, there are few usual treatments, not many clinical trials, and gene therapy options are at an early stage [61]. Natarelli et al. indicated that controlling skin barrier problems and frequent infections is also especially difficult for patients with Netherton syndrome [74]. According to Strober et al., seborrheic dermatitis is challenging because it sometimes comes back, colonization by Malassezia changes, and patients may not stick to their antifungal drugs [54]. Regardless of these diseases, finding treatments that are both safe and work well is difficult for many, which can lead to poor disease control over time.

Recommendation and future direction 

Skin barrier problems are becoming more diverse, necessitating a uniform assessment method. Current methods, such as TEWL and confocal microscopy, are not reliable. Personal medicine and genetic material analysis are driving new methods for restoring the skin barrier. New therapies are being developed to address certain disorders. Future large-scale multi-center randomized controlled trials (RCTs) should be conducted to evaluate the performance and safety of new therapies, such as biologics, small-molecule inhibitors, and gene-based treatments for chronic dermatoses. Studying chronicity, relapse frequency, and life impact can provide a better understanding of these disorders. Further research is needed on genes and molecules involved in these diseases, particularly unusual cases like Netherton syndrome and ichthyosis vulgaris. Researching the skin microbiome and immune system may lead to new targets for medicinal treatments for inflammatory diseases. Real-world studies and cost-effectiveness assessments are crucial for directing clinical and healthcare policies. Patient education and adherence programs can improve treatment results and reduce disease problems.

## Conclusions

Skin barrier dysfunction is a major cause of chronic skin diseases. Advances in barrier biology have led to new medications to improve symptoms and treat the main causes of barrier disease. However, these treatments are not widely used due to unstandardized techniques, sparse long-term data, and unique features. Providing tailored treatments and repairing damaged skin is crucial for better management of chronic dermatologic disorders. Setting diagnostic and treatment guidelines increases clinic efficiency and allows direct comparison between studies.
